# Atmospheric deposition of microplastics in urban, rural, forest environments: A case study of Thulamela Local Municipality

**DOI:** 10.1371/journal.pone.0313840

**Published:** 2025-03-03

**Authors:** Thendo Mutshekwa, Fulufhelo Mulaudzi, Vuledzani P. Maiyana, Lubabalo Mofu, Linton F. Munyai, Florence M. Murungweni

**Affiliations:** 1 Department of Freshwater Invertebrates, Albany Museum, Makhanda, South Africa; 2 Institute of Water Research, Rhodes University, Makhanda (Grahamstown), South Africa; 3 School of Animal, Plant and Environmental Sciences, University of the Witwatersrand, Johannesburg, South Africa; 4 Department of Geography and Environmental Sciences, University of Venda, Thohoyandou, South Africa; 5 Department of Environmental Sciences, College of Agriculture and Environmental Sciences, University of South Africa, Florida Science Campus, Florida, South Africa; 6 South African Institute for Aquatic Biodiversity (SAIAB), Makhanda, South Africa; 7 Department of Ichthyology and Fisheries Science, Rhodes University, Makhanda, South Africa; 8 Aquatic Systems Research Group, School of Biology and Environmental Sciences, University of Mpumalanga, Nelspruit, South Africa; Amity University Amity Institute of Biotechnology, INDIA

## Abstract

Microplastic (MP) pollution, a pressing environmental issue globally, has received considerable attention in marine, freshwater and terrestrial environments. However, studies on the deposition of airborne MPs, particularly in Southern African regions, have received less attention. As such, the current study aimed at investigating the atmospheric deposition of MPs across three different environments, namely urban, rural and forest in the Thulamela Local Municipality, Limpopo, South Africa. We hypothesised that MP deposition will exhibit significant differences between different environments, with the urban environment having the highest MP deposition fluxes due to the dense population in the area and high human activities. The study results showed that MP deposition fluxes varied significantly across the environments, ranging from 90.51 ± 15.19 – 355.64 ± 47.65 particles/m^2^/day, with an overall average of 211.87 ± 31.44 particles/m^2^/day. The highest deposition was recorded in urban environment, while the lowest was observed in the forested environment. The overall deposited MPs were mainly transparent fibres and polyethylene terephthalate (PET). Furthermore, MP deposition found in the forest environment demonstrated a strong positive correlation with rainfall, suggesting that rainfall events play a significant role in the transporting and deposition of MP. The study further shed light on the possible fate of urban and rural atmospheric deposition, which has implications for pristine environments, indicating the transport and potential ecological impacts of MP pollution even in less populated and isolated environments.

## 1. Introduction

Globally, the number of microplastics (MPs) entering the environment in the form of litter or small particles is rapidly growing with projections indicating a total of 25 billion metric tons by the year 2050 [1]. Over the last decade, MP pollution has received much attention due to its extensive environmental impacts and the growing evidence of its presence in aquatic, marine [2] and terrestrial ecosystems [3]. Large plastic particles can degrade into smaller particles, leading to the formation of MPs (less than 5 millimeters in size) [4,5]. These small particles stem from diverse sources, including the breakdown of larger plastic debris into smaller particles [6,7]. Microplastics can find their way into the atmosphere, and once airborne, they can be transported over long distances by wind before eventually settling back to the Earth’s surface [8,9].

Atmospheric deposition of MPs is an emerging environmental issue gaining significant attention since the first discovery by Dris et al. [10]. Atmospheric deposition is defined as the transfer of substances from the atmosphere to the Earth’s surface, comprising both dry and wet deposition, which collectively form the overall atmospheric fallout [11]. Microplastics can readily move from land to air and be carried long distances by winds because of their small size and low density [12–14]. Sources of airborne MPs include industrial emissions [13,15,16]. Few studies have documented the presence of atmospheric MPs in diverse environments, from densely polluted cities [e.g., 17–20] to isolated regions [e.g., 12,14,21,22]. Meteorological conditions exert a profound influence on the transport and deposition of MPs across these diverse environments [23]. For instance, in densely polluted regions with notable vehicular and industrial emissions, meteorological conditions such as wind patterns and temperature can impact the spread and concentration of airborne MPs [24]. Conversely, in isolated regions or less polluted areas, precipitation and wind-driven currents play a crucial role in depositing MPs transported over long distances from urban areas.

Atmospheric MPs can be deposited onto diverse ecosystems, contaminating soil [25,26] and water resources [27], and even entering the food chain through ingestion by various organisms [28]. For instance, MPs can be deposited onto crops [29], potentially entering the food chain and affecting food safety [30]. Furthermore, atmospheric MPs can be inhaled by humans [31], posing health risks, including respiratory issues and potential long-term effects such as inflammation and oxidative stress [32], endocrine disruption [33], and immune system impairment [34]. Susanti et al. [35] and Wu et al. [36] also highlighted the impact of MPs on wildlife since MPs can be ingested by a variety of species, leading to physical harm and exposure to toxic chemicals such as pesticides, heavy metals, and persistent organic pollutants.

Research on atmospheric MPs represented just 0.76% of the overall MP studies by 2021 [37]. In South Africa, MPs studies have focused more on marine [e.g., 38,39] and freshwater environment including rivers [e.g., 40–42], lakes [e.g., 43] and reservoirs [e.g., 44,45]. Studies on atmospheric MPs deposition have been lagging, despite Verster et al. [46] and Verster and Bouwman et al. [47] raising the need to investigate the state of atmospheric MPs in South Africa. As such, the current study was initiated to; 1) investigate the occurrence and characteristics of MPs (across three different environments associated with different human activities and natural processes – urban, rural, and forest environments within Thulamela Local-Municipality, Limpopo, South Africa, 2) examine the relationship between MPs deposition and meteorological variables (humidity, air temperature, wind speed, rainfall) to evaluate the potential role of weather patterns in the distribution and MPs deposition fluxes. The study is based on the hypothesis that MPs deposition fluxes will exhibit significant differences between different environments, with the urban environment having the highest MP deposition fluxes due to the dense population in the area and high human activities and that meteorological variables play a crucial role in the movement and presence of atmospheric MPs. Studying the characteristics of atmospheric MPs in different environments is essential to comprehend the extent of MP contamination and thus contributes to the comprehensive global understanding of atmospheric MPs pollution.

## 2. Methods and materials

### Ethical clearance

Atmospheric MPs deposition samples were collected following ethical clearance approved by the research committee of the University of Venda (Ethical clearance No. FSEA/24/GES/06).

### Study area

The study was conducted in the Thulamela Local-Municipality, situated in the Vhembe District in Limpopo Province over a 6-week sampling period (W1 – W6) from 23 March to 28 April 2024. Three sampling sites were selected to represent distinct different environments. Site 1 (22°52′47.3ʺS; 30°30′34.8ʺE – hereafter referred to as urban), located in the town of Thohoyandou, represents an environment with high population density, significant economic activity, substantial infrastructure development and human settlement. Site 2 (22°57′16.5ʺS; 30°22′11.4ʺE – hereafter referred to as rural), located in the village of Duthuni, represents an environment with low population density, scattered settlements, limited access to amenities and a slower pace of life. Site 3 (22°53′08.9ʺS; 30°18′39.9ʺE – hereafter referred to as forest), located in the Thathe Vondo Forest in the Soutpansberg Mountains, represents a natural environment dominated by dense vegetation, minimal human presence, and limited infrastructure. The forest is surrounded by tall and diverse trees, which are multi-layered, and have a well-developed canopy and understory layers [48]. The forest hosts trees such as *cryptocarya liebertiana*, *Drypetes gurrardii*, *Rawsonia lucida*, *Combretum krausii*, *Ficus natalensis*, *Podocarpus latifolius*, *Xymalos monospora*, *Cussonia spicata* and *Oxyathus gerrardii* [48]. The climatic conditions vary considerably within the Thulamela local municipality. The mean air temperature ranges from 18 °C to 28°C with an average of about 23 °C, whereas the mean rainfall ranges from 450 mm to 750 mm, with an average of about 600 mm.

### Sampling and separation of MPs

Meteorological variables (humidity, air temperature, windspeed, rainfall), were obtained from the South African Weather Service (measurement frequency, 4 h; [49]) and nearby weather stations. A total of 54 deposition samples (3 sites × 3 replicates × 6 weeks (including both wet deposition and dry deposition)) were passively collected using a 25 L steel bucket (diameter 305mm; height 400mm) following a procedure by Sun et al. [50]. The sampling points in the urban environment included the University of Venda, a residential area near the main road and an open space near Thavhani Mall. The sampling points in the rural environment included an open football field, a residential area near the main road and an agricultural field. The sampling points in the forest environment included open space inside the forest, open space near an infrastructure and gravel road. Buckets were marked and placed at a fixed point, 2 meters above ground, following recommendations by Klein et al. [19], to avoid contamination from ground-level sources. The buckets were left open to the atmosphere for 24 h, to collect airborne particles. After the exposure, the buckets were carefully closed with aluminium foil, transported back to the laboratory and treated immediately. In the laboratory, the samples were treated with 30% H_2_O_2_ to remove organic materials and then filtered through 1000, 500, 250 and 100 μm sieves. To remove the adhesion of particles to the internal walls of buckets, the buckets were washed with MilliQ water three times. The sieves were dried at 30°C overnight prior to MP identification under a dissecting microscope. The deposition flux of MPs was determined by considering the quantity of MPs detected (particles), the surface area of the sample bucket opening (m^2^), and the sampling period (day). Flux values were expressed as particles/m^2^/day, representing the count of MPs deposited per square meter per day.

Fourier Transform Infrared Spectroscopy (FTIR) analyses were also used to confirm visual inspections of MP particles [51]. MP particles (30%) were selected for polymer identification through vibrational Platinum-ATR Fourier-transform infrared spectroscopy using the Bruker Alpha model from Germany. The technique covered a spectral range of 650–4000 cm^ −1^, had a resolution of 8 cm^ −1^, and conducted 16 scans per analysis. Prior to each sample analysis, background scans were performed, and the ATR crystal was cleaned with 70% propanol. All obtained spectra scans were compared and verified against the following databases: Hummel Polymer Sample Library, HR Polymer Additives and Plasticizers, HR Hummel Polymers and Additives, and Synthetic Fibres by Microscope.

### Quality assurance and quality control

Stringent quality assurance protocols were implemented to prevent background contamination. Personnel wore cotton lab coats, masks, and nitrile gloves during the procedures. Plastic equipment and tools were shielded with fresh tin foil when not in operation. The MilliQ water used in the tests underwent filtration. Glassware such as petri dishes and filtering devices, as well as stainless-steel tools, were rinsed with distilled water. Filtration and identification of MP particles occurred within a laminar flow hood cabinet to restrict airborne contamination. To determine whether contamination was a confounding factor under laboratory conditions, blanks of glass microfiber filters were situated as follows: (*i*) open inside the laminar; (*ii*) open in the oven and (*iii*) open in the laboratory next to the microscope, following procedure outlined by Davison and Asch [52]. Laboratory blanks were used as “negative control” to cross-check the contamination of plastic materials. Extraction efficiencies were assessed by filtering known quantities of particles to verify the recovery rates of MP particles, following recommendations of Dimante-Deimantovica et al. [53]. The recovery process demonstrated an efficiency rate exceeding 95%.

### Statistical analyses

A two-way analysis of variance (ANOVA) was used to assess any MP particle differences across sites (urban, rural, and forest) after testing for homogeneity of variances (Levene’s test, *p* > 0.05) and normality of distribution (Shapiro-Wilk test, *p* > 0.05). Significant variables were further assessed for pairwise comparisons using Tukey’s post hoc analysis. Pearson correlations were also carried out to assess the relationships between meteorological parameters and MP particle numbers. In all analyses, significance was inferred at *p* < 0.05 and all statistical analyses were performed using IBM SPSS version 25.

## 3. Results

### Microplastic deposition fluxes

**Fig 1:**
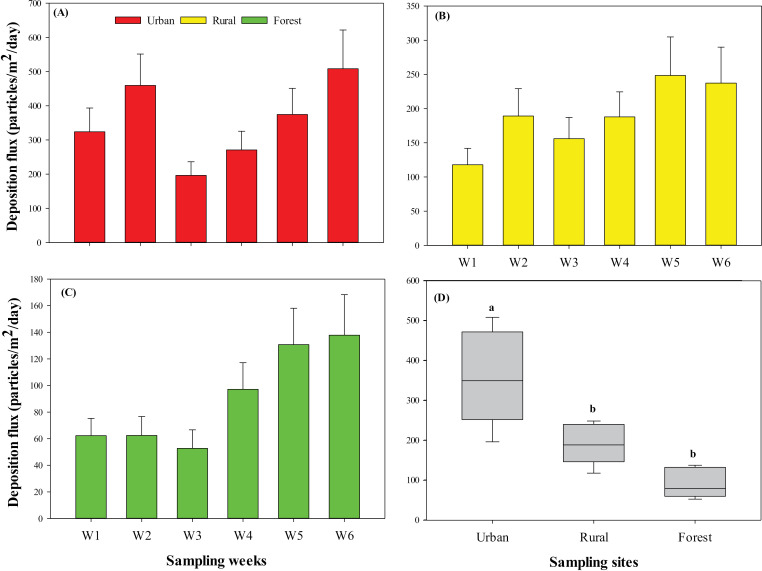
Microplastic deposition fluxes (Mean ± SD) collected in (A) urban, (B) rural, and (C) forest over time (6 weeks); (D) Boxplot highlight significance analysis across sampling sites in the Thulamela Local Municipality, Limpopo, South Africa.

Microplastics were found across all three sampling environments (Fig 1; Table in S1 Table). Overall, across environments, MPs deposition had an overall average of 211.87 ± 31.44 particles/m^2^/day, ranging from 52.70 ± 13.94 particles/m^2^/day to 508.46 ± 113.24 particles/m^2^/day. The highest MPs deposition was found in urban (Site 1: 508.46 ± 113.24 particles/m^2^/day; Fig 1A) during week 6, whereas the lowest MPs deposition was recorded in the forest (Site 3: 52.70 ± 13.94 particles/m^2^/day; Fig 1C) during week 3 (Fig 1; Table in S1 Table). Microplastic deposition fluxes differed significantly among the three environments (F = 17.278; *p* < 0.001; Fig 1D). Overall, across weeks, urban exhibited the highest average MPs deposition (355.64 ± 47.65 particles/m^2^/day), followed by rural (189.46 ± 20.0 particles/m^2^/day) and forest (90.51 ± 15.19 particles/m^2^/day) (Fig 1D; Table in S1 Table). No significant differences were observed among weeks within each site (*p* > 0.05).

### Microplastics colour, shape, size and polymer type

**Fig 2:**
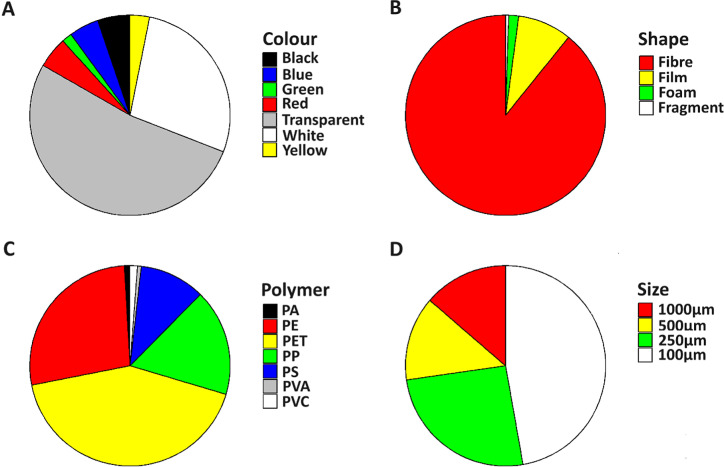
The proportion of fibres (A) colour, (B) shape, (C) polymer type, and (D) size found across three sampling sites (urban, rural, forest) in the Thulamela Municipality, Vhembe District, Limpopo, South Africa.

A variety of colours were observed in the deposited MPs collected across sites and weeks (Fig 2A; 4). Transparent colour was the predominant colour, accounting for 52.4% of all the MPs collected. White and black MPs were the second and third most abundant colours, accounting for 27.8% and 5.2%, respectively (Fig 2A; Table in S2 Table). Red (5.0%), blue (4.8%), yellow (3.1%) and green (1.6%) were other colours found. In terms of sites, transparent was predominant in urban (57.6%) and forest (60.3%), whereas in rural, white was the dominant colour (44.6%).

Four MP shapes were identified, with fibres being the most dominant (89.2%) (Fig 2B; Table in S2 Table). Fragment, foam and film were also observed, accounting for 8.7%, 1.6% and 0.5%, respectively, of the total count (Fig 2B). Following Fourier-transform infrared spectroscopy (FT-IR) analysis of the selected MP particles, seven polymer types were found, i.e., polyamide (PA), polyethylene (PE), polyethylene terephthalate (PET), polypropylene (PP), polystyrene (PS), polyvinyl alcohol (PVA), polyvinyl chloride (PVC). Polyethylene terephthalate (42.3%) was found to be the dominant polymer type, followed by PE (27.2%), PP (17.2%), PS (10.6%), PVC (1.2%), PA (0.9%) and PVA (0.6%) (Fig 2C; S2 Table). Microplastics were also found in different sieve sizes (see methods and materials). Most MPs were found in 100 μm sieve size across Urban, Rural and Forest (range, 41.3% – 58.1%). Microplastics were also found in 250 μm (21.5% – 28.3%), 500 μm (7.3% – 17.8%), and 1000 μm (12.3% – 14.6%) (Fig 2D; Table in S2 Table).

### Relationship between meteorological variables and MPs deposition

**Fig 3:**
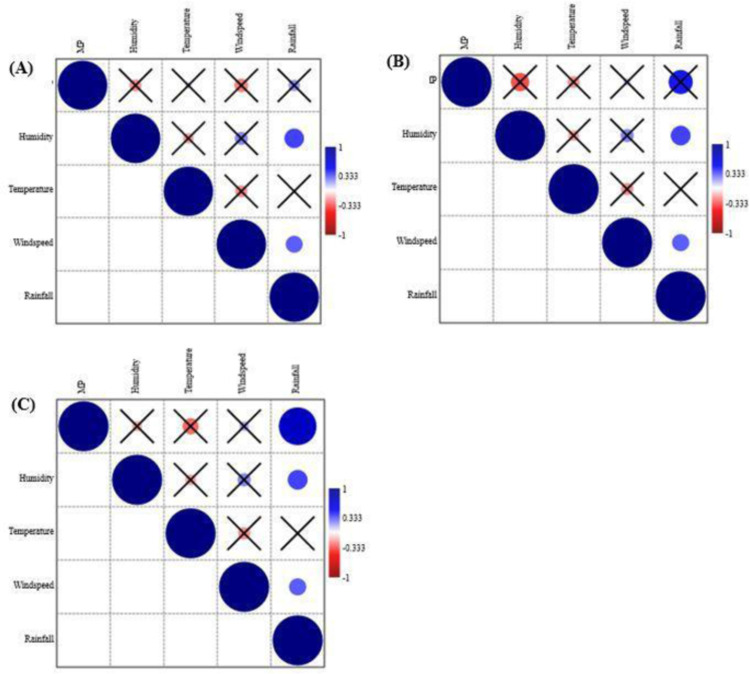
Pearson correlations between meteorological variables and (A) urban, (B) rural and (C) forest environment (*p* > 0.05 crossed).

Table in S3 Table indicates the range of meteorological variables measured across the three environments (urban, rural, and forest) over six weeks. Based on Pearson correlation analysis, there was no significant relationship (*p* > 0.05) between meteorological variables and MPs deposition for urban and rural (Fig 3A, 3B). However, a significant relationship was observed between rainfall and forest (r = 0.74; *p* = 0.005; Fig 3C), whereas humidity, air temperature and wind speed highlighted a non-significant relationship (*p* > 0.05) (Fig 3C).

## 4. Discussion

In this study, atmospheric MPs deposition was investigated in the Thulamela Local Municipality, Limpopo, South Africa, to contribute to the global understanding of MPs deposition and to emphasize the need for atmospheric MPs deposition studies in Southern Africa as suggested by Verster and Bouwman et al. [47]. This study investigated atmospheric MPs deposition in the Thulamela Local Municipality across three different environments, i.e., urban, rural, and forest. Overall, our findings showed that atmospheric MPs deposited in the urban was significantly higher than MPs deposited in rural and forest environments. We also found that MPs colour, shape, size and polymer type were consistent across the three different environments, with transparent, fibres, PET and smaller sized particles accounting for the highest proportions. No significant correlations were observed between meteorological variables (humidity, air temperature, wind speed, and rainfall) and MP deposition fluxes for urban and forest, whereas a positive significant relationship observed for rainfall and forest environment.

Table 1 indicates the atmospheric deposition of MPs found around the world in different environments. Our findings corroborated with the findings of a few existing studies around the world that reported higher MPs deposition in urban environments, compared to rural and forest/remote [e.g., 17,22; Table 1]. Zhang et al. [22] collected MPs deposition across Beijing City, China and found variation in MPs deposition across urban (395.07 ± 41.44 particles/m^2^/day), rural (180.12 ± 42.22 particles/m^2^/day) and forest (133.18 ± 47.44 particles/m^2^/day) which corresponded with our findings. Some studies, however, have reported significantly lower MP depositions in urban environments compared to our results. Dris et al. [17] collected MPs deposition on the rooftop in an urban/suburban environment in Paris, France and found MPs deposition fluxes ranging from 53 ± 38 to 110 ± 96 particles/m^2^/day. Klein et al. [19] collected MPs deposition in the urban environment of Northern Germany, Germany and found MPs deposition ranging from 53 to 166 particles/m^2^/day. The study in Dongguan City, China by Cai et al. [18] also found MP deposition of 36 particles/m^2^/day, which was ten times lower than our findings. Our findings for rural environment showed lower MPs deposition than those reported by Li et al. [54] in Quzhou County, Hebei Province, China (86 – 75,421 particles/m^2^/day) but higher than those reported by Zhang et al. [22] in Beijing city, China (180.12 ± 42.22 particles/m^2^/day). The forest environment, typically less populated and more isolated, is often associated with lower MPs deposition [56]. This was evident in our study, where the forest environment recorded the lowest MP deposition fluxes. In comparison with other studies, MPs deposition in the forest environment was lower than those reported by Allen et al. [12] in Pyrenees, France (133.18 ± 47.44 particles/m^2^/day), Brahney et al. [55] in Protected areas, United States (132 ± 6 particles/m^2^/day).

**Table 1:** Microplastic deposition fluxes from the atmosphere in the current study and around the world.

Country	Environmental type	Sampling height from the ground (m)	Sampling methods	MP shapes found	MP found (particles/m^2^/day)	Reference
1. Beijing City, China	Urban Rural Forest	3–5	Passive samplers	Fibres (86.27%) Fragment (7.38%), Films (5.12%), Others	395.07 ± 41.44 180.12 ± 42.22 133.18 ± 47.44	[22]
2. Dongguan, China	Urban		Passive samplers	Fiber (90.1%), Fragment (6.8%), Film (2.9%), Foam(0.2%)	36 ± 7	[18]
3. Thulamela Local Municipality, Limpopo, South Africa	Urban Rural Forest	2	Passive samplers	Fibres (89.2% Fragments (8.7%), Films (1.6%) Foams (0.5%)	355.6 ± 47.6 189.5 ± 20.0 90.5 ± 15	Current study
4. Northern Germany, Germany	Urban Suburban Rural	2	Active pump samplers	Fibres Fragments	53 to 166	[19]
5. Quzhou County, Hebei Province, China	Rural	1	Passive samplers		86 to 75,421	[54]
6. Paris, France	Urban Suburban	rooftop	Passive samplers	Fiber (the only targeted shape)	110 ± 96 53 ± 38	[17]
7. Pyrenees, France	Remote	3–5	Passive samplers	Fragment (68%), Fiber (12%), Film (20%)	365 ± 69	[12]
8. Protected areas, United States of America	Rural/remote		_	Fibres, particles	132 ± 6	[55]

The differences in MP deposition fluxes between studies may be attributed to varying sources of MPs across different countries, cities, and environments [57]. These variations are influenced by a multitude of factors, including the types of plastic products used, industrial activities, waste management practices, population density, proximity to water bodies, local weather patterns, the presence of manufacturing facilities, agricultural practices, waste collection systems, and environmental policies. As highlighted by Dris et al. [58], urban environments with higher population density, vehicle emissions, urbanisation and industrialisation generally experience higher MP deposition fluxes than less populated and remote environments. This is due to the higher prevalence of MP emissions in urban areas, where anthropogenic activities like textile production, packaging, construction, and industrial emissions significantly contribute to the sources of MPs [58]. This may explain the high MPs deposition observed in the urban environment in our study, which could pose potential risks to the locals of Thohoyandou town and animals through inhalation, ingestions, and transfer of toxic substances [59]. Our findings also highlight the potential contamination of various environments, including reservoirs, rivers, and other freshwater systems, posing a threat to aquatic organisms due to the ingestion, entanglement and toxicological effects associated with MPs [60]. The current study is crucial in comprehending the extent of MPs pollution and can help inform targeted conservation efforts and policy development aimed at reducing MPs contamination [60].

We also found MPs in different colours and sizes. Transparent was the most dominant colour, followed by white and black, whereas MPs sized 100 μm were the most dominant. Similarly, Sun et al. [50] attested that transparent MPs in different environmental compartments are likely to lose their original colour over time. This colour fading may be attributed to exposure to environmental factors such as sunlight, air, and water, which contribute to the degradation of plastic particles [61]. The prevalence of smaller-sized MPs, particularly fibres, may be due to their low density and aerodynamic properties. These characteristics enable them to remain airborne longer and disperse more widely [62].

Consistent with other existing studies [e.g., 10,18,22,55,63,64] fibres were the most MPs shape found in the current study, accounting for 89.2% of the total count. This also explains the domination of PET, PE and PP found in the current study. These materials are widely used in the textile industry (for instance, clothing, home furnishing, industrial applications, packaging and household products) due to their versatility, durability, and affordability [65]. Thohoyandou town, which is in the urban environment is known to have high vehicle and foot traffic density [66]. Tire abrasion from vehicles can generate fragments and fibres, as these polymers are incorporated into certain textile reinforcements and other components of the tire structure, including outer coatings. Additionally, plastic waste produced from foot traffic during shopping activities can degrade over time into MPs and eventually enter the atmosphere. In rural environments, where agricultural activities and settlements are prevalent, agricultural products and laundry effluent can release certain polymers into the air, which may be the primary source of the polymers we identified. The Thathe Forest in our study, which attracts tourists and recreational activities, may have introduced some of the MPs we found due to illegal dumping and littering. Furthermore, PE, PP, and PVC, which were identified in the current study, are materials commonly used in the production of fishing gear. This suggests they may be another source of MPs in the rural and forest environments, particularly since these environments are near the Mutshundudi River, Lake Fundunzi and Thathe Vondo Dam, which are popular fishing locations. Over time, plastic particles from fishing gear can degrade and become airborne MPs.

As highlighted by Szewc et al. [67], the transport and deposition of MPs in the atmosphere are influenced by meteorological conditions such as rainfall, and wind direction. Meteorological conditions and MPs deposition rates for each sampling site and date are provided in S1 and S3 Tables. In the current study, no significant relationship between meteorological variables and MP deposition was found in urban and rural environments, suggesting that the local sources exert a significant impact on the deposition of MPs. Similar findings were observed in the study carried out by Wright et al. [63], which showed a negative effect on atmospheric deposition of MPs in central London. A significant relationship was found between rainfall and MP deposition fluxes in the forest environment. As reported by Zhang et al. [15], rainfall events can promote the deposition of atmospheric MPs. In the forest environment, lowest levels of MPs particles were found among the weeks with the least rainfall (e.g., week 1–3), whereas high MP particles were found during high rainfall (e.g., week 6; 137.8 particles/m^2^/day). Our findings suggest that rainfall events can influence the deposition of atmospheric MP in remote and isolated environments. As indicated by Roblin et al. [68] factors affecting deposition of atmospheric MP are probably complex.

Across all the three environments, the urban environment in Thohoyandou was the most polluted by atmospheric MPs. The potential local sources of MPs were probably from intense vehicle emissions, waste disposal and high human activities. This town is also home to Thohoyandou Wastewater treatment plant, landfill, and two shopping malls, which can release a significant number of MPs into the atmosphere. Variations in MP deposition found in the current study and other similar studies might be attributed to different sampling heights above ground level and sampling methods, such discrepancies can influence the concentration and types of MPs deposited, leading to inconsistent results. To address this issue, there is a need for standardized height and sampling protocols in atmosphere MPs deposition. Establishing uniform guidelines for the height at which MPs deposition is collected and the methods used will ensure more comparable and reliable findings, intimately improving our global understanding of atmospheric MPs pollution. The findings of this study establish a fundamental reference point for the quantity of MPs in the atmosphere of Southern Africa and highlight the importance of investigating the transport, source, distribution and potential effects, highlighting the necessity for further research in this area.

## 5. Conclusion

In the current study, we reported evidence of atmospheric MPs pollution in the Thulamela Local Municipality, Limpopo, South Africa. A significant difference in MP deposition fluxes was found across urban, rural and forest environments, with the highest average MP deposition of 355.64 ± 47.65 particles/m^2^/day found in urban environment and the lowest average MP deposition of 90.51 ± 15.19 particles/m^2^/day found in a forest environment. Transparent fibres, PET and small sized MPs accounted for a large proportion of the total MP number found. Atmospheric MPs deposition corresponded with anthropogenic activities around each environment, with the urban environment associated with high population density, high human activities and emission sources. We also found significant relationship between forest environment and rainfall, which corresponds to the amount of MPs found between the weeks. Although the current study was carried out over six weeks period, more long-term monitoring using both active and passive sampling methods is recommended for further studies to enhance the understanding of atmospheric MPs deposition and influencing factors. Furthermore, future studies should also investigate other meteorological variables in order to enhance the understanding of the influence of weather patterns on MPs deposition. In general, studies on atmospheric deposition of MPs in Southern Africa should receive increased attention, given the fact that these airborne particles can settle in marine, freshwater and terrestrial ecosystems, thus impacting a wide range of ecological processes and potentially entering food webs, posing risks to wildlife and human health.

## Supporting information

S1 Table Microplastic deposition fluxes across the three sampling environments over time (6 weeks). (DOCX)

S2 Table Overall colour, shape, polymer and size proportions (%) across the three environments over time (6 weeks). (DOCX)

S3 Table Range of meteorological variables measured across the three sampling environments over time (6 weeks). (DOCX)

## References

[1] Plastics Europe. Plastics - the facts 2022. An analysis of European plastics production, demand, conversion and end-of-life management. 2022 [cited 23 Apr 2024]. Available from: https://plasticseurope.org/knowledge-hub/plastics-the-facts-2022/PE-PLASTICS-THE-FACTS_FINAL_DIGITAL-1.pdf

[2] Tang Y, Liu Y, Chen Y, Zhang W, Zhao J, He S, et al. A review: Research progress on microplastic pollutants in aquatic environments. Sci Total Environ. 2021;766:142572. doi: 10.1016/j.scitotenv.2020.142572 33183825

[3] Surendran U, Jayakumar M, Raja P, Gopinath G, Chellam PV. Microplastics in terrestrial ecosystem: Sources and migration in soil environment. Chemosphere. 2023;318:137946. doi: 10.1016/j.chemosphere.2023.137946 36708782

[4] Arthur C, Baker JE, Bamford HA. Proceedings of the International Research Workshop on the Occurrence, Effects, and Fate of Microplastic Marine Debris, September 9-11, 2008, University of Washington Tacoma, Tacoma, WA, USA.

[5] Andrady AL. The plastic in microplastics: A review. Mar Pollut Bull. 2017;119(1):12–22. doi: 10.1016/j.marpolbul.2017.01.082 28449819

[6] Duis K, Coors A. Microplastics in the aquatic and terrestrial environment: sources (with a specific focus on personal care products), fate and effects. Environ Sci Eur. 2016;28(1):2. doi: 10.1186/s12302-015-0069-y 27752437 PMC5044952

[7] Bhat MA, Gedik K, Gaga EO. A preliminary study on the natural aging behavior of microplastics in indoor and outdoor environments. Int J Environ Sci Technol. 2024;21(2):1923–36. doi: 10.1007/s13762-023-05319-4

[8] Sathyamohan G, Sewwandi M, Ambade B, Vithanage M. Sources and circulation of microplastics in the aerosphere–atmospheric transport of microplastics. Microplasti Ecosphere. 2023;125–46. doi: 10.1002/9781119879534.ch8

[9] Belioka MP, Achilias DS. How plastic waste management affects the accumulation of microplastics in waters: a review for transport mechanisms and routes of microplastics in aquatic environments and a timeline for their fate and occurrence (past, present, and future). Water Emer Contam Nanoplast. 2024;3(2). 10.20517/wecn.2024.09

[10] Dris R, Gasperi J, Rocher V, Saad M, Renault N, Tassin B. Microplastic contamination in an urban area: a case study in Greater Paris. Environ Chem. 2015;12(5):592–9. doi: 10.1071/EN14167

[11] Hupfer P, Kuttler W. Witterung und Klima. Eine Einführung in die Meteorologie und Klimatologie. 2005;11.

[12] Allen S, Allen D, Phoenix VR, Le Roux G, Durántez Jiménez P, Simonneau A, et al. Atmospheric transport and deposition of microplastics in a remote mountain catchment. Nat Geosci. 2019;12(5):339–44. doi: 10.1038/s41561-019-0335-5

[13] Chen G, Feng Q, Wang J. Mini-review of microplastics in the atmosphere and their risks to humans. Sci Total Environ. 2020;703:135504. doi: 10.1016/j.scitotenv.2019.135504 31753503

[14] Evangeliou N, Grythe H, Klimont Z, Heyes C, Eckhardt S, Lopez-Aparicio S, et al. Atmospheric transport is a major pathway of microplastics to remote regions. Nat Commun. 2020;11(1):3381. doi: 10.1038/s41467-020-17201-9 32665541 PMC7360784

[15] Zhang Y, Kang S, Allen S, Allen D, Gao T, Sillanpää M. Atmospheric microplastics: A review on current status and perspectives. Earth-Sci Rev. 2020;203:103118. doi: 10.1016/j.earscirev.2020.103118

[16] O’Brien S, Rauert C, Ribeiro F, Okoffo ED, Burrows SD, O’Brien JW, et al. There’s something in the air: a review of sources, prevalence and behaviour of microplastics in the atmosphere. Sci Total Environ. 2023;874:162193. doi: 10.1016/j.scitotenv.2023.162193 36828069

[17] Dris R, Gasperi J, Saad M, Mirande C, Tassin B. Synthetic fibers in atmospheric fallout: a source of microplastics in the environment? Mar Pollut Bull. 2016;104(1–2):290–3. doi: 10.1016/j.marpolbul.2016.01.006 26787549

[18] Cai L, Wang J, Peng J, Tan Z, Zhan Z, Tan X, et al. Characteristic of microplastics in the atmospheric fallout from Dongguan city, China: preliminary research and first evidence. Environ Sci Pollut Res Int. 2017;24(32):24928–35. doi: 10.1007/s11356-017-0116-x 28918553

[19] Klein M, Bechtel B, Brecht T, Fischer EK. Spatial distribution of atmospheric microplastics in bulk-deposition of urban and rural environments–A one-year follow-up study in northern Germany. Sci Total Environ. 2023;901:165923. doi: 10.1016/j.scitotenv.2023.165923 37532052

[20] Wang C, Guo M, Yan B, Wei J, Liu F, Li Q, et al. Characteristics of microplastics in the atmosphere of Anyang City. Environ Monit Assess. 2024;196(4):350. doi: 10.1007/s10661-024-12493-6 38460005

[21] Trainic M, Flores JM, Pinkas I, Pedrotti ML, Lombard F, Bourdin G, et al. Airborne microplastic particles detected in the remote marine atmosphere. Commun Earth Environ. 2020;1(1):64. doi: 10.1038/s43247-020-00061-y

[22] Zhang R, Jia X, Wang K, Lu L, Li F, Li J, et al. Characteristics, sources and influencing factors of atmospheric deposition of microplastics in three different ecosystems of Beijing, China. Sci Total Environ. 2023;883:163567. doi: 10.1016/j.scitotenv.2023.163567 37094671

[23] Munyaneza J, Jia Q, Qaraah FA, Hossain MF, Wu C, Zhen H, et al. A review of atmospheric microplastics pollution: In-depth sighting of sources, analytical methods, physiognomies, transport and risks. Sci Total Environ. 2022;822:153339. doi: 10.1016/j.scitotenv.2022.153339 35077799

[24] Sridharan S, Kumar M, Singh L, Bolan NS, Saha M. Microplastics as an emerging source of particulate air pollution: A critical review. J Hazard Mater. 2021;418:126245. doi: 10.1016/j.jhazmat.2021.126245 34111744

[25] Wang J, Liu X, Li Y, Powell T, Wang X, Wang G, et al. Microplastics as contaminants in the soil environment: A mini-review. Sci Total Environ. 2019;691:848–57. doi: 10.1016/j.scitotenv.2019.07.209 31326808

[26] Zhang Z, Zhao S, Chen L, Duan C, Zhang X, Fang L. A review of microplastics in soil: Occurrence, analytical methods, combined contamination and risks. Environ Pollut. 2022;306:119374. doi: 10.1016/j.envpol.2022.119374 35490998

[27] Bhat MA, Janaszek A. Evaluation of potentially toxic elements and microplastics in the water treatment facility. Environ Monit Assess. 2024;196(5):475. doi: 10.1007/s10661-024-12651-w 38662271 PMC11045652

[28] Bajt O. From plastics to microplastics and organisms. FEBS Open Bio. 2021;11(4):954–66. doi: 10.1002/2211-5463.13120 33595903 PMC8016121

[29] Dhevagi P, Keerthi Sahasa RG, Poornima R, Ramya A. Unveiling the effect of microplastics on agricultural crops–a review. Int J Phytoremediation. 2024;26(6):793–815. doi: 10.1080/15226514.2023.2275152 37941363

[30] Lehel J, Murphy S. Microplastics in the food chain: food safety and environmental aspects. Rev Environ Contam Toxicol. 2021;259:1–49. doi: 10.1007/398_2021_77 34611754

[31] Prata JC. Airborne microplastics: consequences to human health? Environ Pollut. 2018;234:115–26. doi: 10.1016/j.envpol.2017.11.043 29172041

[32] Köktürk M, Özgeriş FB, Atamanalp M, Ucar A, Özdemir S, Parlak V, et al. Microplastic-induced oxidative stress response in turbot and potential intake by humans. Drug Chem Toxicol. 2024;47(3):296–305. doi: 10.1080/01480545.2023.2168690 36656072

[33] Segovia‐Mendoza M, Nava‐Castro KE, Palacios‐Arreola MI, Garay‐Canales C, Morales‐Montor J. How microplastic components influence the immune system and impact on children health: Focus on cancer. Birth Defect Res. 2020;112(17):1341–61. doi: 10.1002/bdr2.1779 32767490

[34] Remmelts M. Effect of nano-and microplastics on the human immune system and their influence on inflammatory bowel disease (Doctoral dissertation). 2021.

[35] Susanti NK, Mardiastuti A, Wardiatno Y. Microplastics and the impact of plastic on wildlife: a literature review. InIOP Conference Series: Earth and Environmental Science 2020 Jul 1 (Vol. 528, No. 1, p. 012013). IOP Publishing.

[36] Wu T, Shu X, Wang C, Li W, Zhu D, Wang J, et al. Microplastic pollution of threatened terrestrial wildlife in nature reserves of Qinling Mts., China. Global Ecol Conserv. 2024;51:e02865. doi: 10.1016/j.gecco.2024.e02865

[37] Alfonso MB, Arias AH, Ronda AC, Piccolo MC. Continental microplastics: Presence, features, and environmental transport pathways. Sci Total Environ. 2021;799:149447. doi: 10.1016/j.scitotenv.2021.149447 34371405

[38] Nel HA, Froneman PW. A quantitative analysis of microplastic pollution along the south-eastern coastline of South Africa. Mar Pollut Bull. 2015;101(1):274–9. doi: 10.1016/j.marpolbul.2015.09.043 26433774

[39] Naidoo T, Glassom D. Sea-surface microplastic concentrations along the coastal shelf of KwaZulu–Natal, South Africa. Mar Pollut Bull. 2019;149:110514. doi: 10.1016/j.marpolbul.2019.110514 31546106

[40] Nel HA, Dalu T, Wasserman RJ. Sinks and sources: Assessing microplastic abundance in river sediment and deposit feeders in an Austral temperate urban river system. Sci Total Environ. 2018;612:950–6. doi: 10.1016/j.scitotenv.2017.08.298 28886547

[41] Dalu T, Banda T, Mutshekwa T, Munyai LF, Cuthbert RN. Effects of urbanisation and a wastewater treatment plant on microplastic densities along a subtropical river system. Environ Sci Pollut Res Int. 2021;28(27):36102–11. doi: 10.1007/s11356-021-13185-1 33686602 PMC8277625

[42] Saad D, Ndlovu M, Ramaremisa G, Tutu H. Microplastics in freshwater environment: the first evaluation in sediment of the Vaal River, South Africa. Heliyon. 2022;8(10):e11118. doi: 10.1016/j.heliyon.2022.e11118 36339993 PMC9634034

[43] Nava V, Chandra S, Aherne J, Alfonso MB, Antão-Geraldes AM, Attermeyer K, et al. Plastic debris in lakes and reservoirs. Nature. 2023;619(7969):317–22. doi: 10.1038/s41586-023-06168-4 37438590

[44] Mbedzi R, Cuthbert RN, Wasserman RJ, Murungweni FM, Dalu T. Spatiotemporal variation in microplastic contamination along a subtropical reservoir shoreline. Environ Sci Pollut Res Int. 2020;27(19):23880–7. doi: 10.1007/s11356-020-08640-4 32301080

[45] Mutshekwa T, Munyai LF, Mugwedi L, Cuthbert RN, Dondofema F, Dalu T. Seasonal occurrence of microplastics in sediment of two South African recreational reservoirs. Water Biol Security. 2023;2(3):100185. doi: 10.1016/j.watbs.2023.100185

[46] Verster C, Minnaar K, Bouwman H. Marine and freshwater microplastic research in South Africa. Integr Environ Assess Manag. 2017;13(3):533–5. doi: 10.1002/ieam.1900 28440929

[47] Verster C, Bouwman H. Contextualization of airborne microplastic pollution in the South African environment. In Proceedings of the 2018 Conference of the National Association for Clean Air 2018.

[48] Sinthumule NI, Mashau ML. Traditional ecological knowledge and practices for forest conservation in Thathe Vondo in Limpopo Province, South Africa. Global Ecol Conserv. 2020;22:e00910. doi: 10.1016/j.gecco.2020.e00910

[49] South African Weather Service (SAWS). Department of Forestry, Fisheries and the Environment. Available from: https://www.weathersa.co.za/ [cited 1 May 2024].

[50] Sun J, Peng Z, Zhu ZR, Fu W, Dai X, Ni BJ. The atmospheric microplastics deposition contributes to microplastic pollution in urban waters. Water Res. 2022;225:119116. doi: 10.1016/j.watres.2022.119116 36152440

[51] Mintenig SM, Löder MG, Primpke S, Gerdts G. Low numbers of microplastics detected in drinking water from ground water sources. Sci Total Environ. 2019;648:631–5. doi: 10.1016/j.scitotenv.2018.08.178 30121540

[52] Davison P, Asch RG. Plastic ingestion by mesopelagic fishes in the North Pacific Subtropical Gyre. Mar Ecol Prog Ser. 2011;432:173–80. doi: 10.3354/meps09142

[53] Dimante-Deimantovica I, Suhareva N, Barone M, Putna-Nimane I, Aigars JH. Threshold values and contribution towards better understanding of recovery rate in microplastic research. MethodsX. 2022;9:101603. doi: 10.1016/j.mex.2021.101603 34976752 PMC8683728

[54] Li J, Zhang J, Ren S, Huang D, Liu F, Li Z, et al. Atmospheric deposition of microplastics in a rural region of North China Plain. Sci Total Environ. 2023;877:162947. doi: 10.1016/j.scitotenv.2023.162947 36940745

[55] Brahney J, Hallerud M, Heim E, Hahnenberger M, Sukumaran S. Plastic rain in protected areas of the United States. Science. 2020;368(6496):1257–60. doi: 10.1126/science.aaz5819 32527833

[56] Welsh B, Aherne J, Paterson AM, Yao H, McConnell C. Atmospheric deposition of anthropogenic particles and microplastics in south-central Ontario, Canada. Sci Total Environ. 2022;835:155426. doi: 10.1016/j.scitotenv.2022.155426 35469860

[57] Padha S, Kumar R, Dhar A, Sharma P. Microplastic pollution in mountain terrains and foothills: A review on source, extraction, and distribution of microplastics in remote areas. Environ Res. 2022;207:112232. doi: 10.1016/j.envres.2021.112232 34687754

[58] Dris R, Gasperi J, Tassin B. Sources and fate of microplastics in urban areas: a focus on Paris megacity. Freshwater microplastics: emerging environmental contaminants? 2018. p. 69–83.

[59] Islam MZ, Zaman SU, Sami NI, Roy S, Jeba F, Islam MS, et al. Human inhalation exposure assessment of the airborne microplastics from indoor deposited dusts during winter in Dhaka, Bangladesh. Heliyon. 2024;10(16):e36449. doi: 10.1016/j.heliyon.2024.e36449 39253203 PMC11381766

[60] Thacharodi A, Hassan S, Meenatchi R, Bhat MA, Hussain N, Arockiaraj J, et al. Mitigating microplastic pollution: A critical review on the effects, remediation, and utilization strategies of microplastics. J Environ Manage. 2024;351:119988. doi: 10.1016/j.jenvman.2023.119988 38181686

[61] Bertoldi C, Lara LZ, Mizushima FA, Martins FC, Battisti MA, Hinrichs R, et al. First evidence of microplastic contamination in the freshwater of Lake Guaíba, Porto Alegre, Brazil. Sci Total Environ. 2021;759:143503. doi: 10.1016/j.scitotenv.2020.143503 33218802

[62] Baron PA. Measurement of Airborne Fibers A Review. Ind Health. 2001;39(2):39–50. doi: 10.2486/indhealth.39.39 11341557

[63] Wright SL, Ulke J, Font A, Chan KL, Kelly FJ. Atmospheric microplastic deposition in an urban environment and an evaluation of transport. Environ Int. 2020;136:105411. doi: 10.1016/j.envint.2019.105411 31889555 PMC7013824

[64] Dong H, Wang L, Wang X, Xu L, Chen M, Gong P, et al. Microplastics in a remote lake basin of the Tibetan Plateau: Impacts of atmospheric transport and glacial melting. Environ Sci Technol. 2021;55(19):12951–60. doi: 10.1021/acs.est.1c03227 34524792

[65] Preston J. Man-made fibre. Encyclopædia Britannica. 2016 [cited 16 Oct 2024]. Available from: www.britannica.com/technology/man-made-fiber

[66] Novela RJ, Gitari WM, Chikoore H, Molnar P, Mudzielwana R, Wichmann J. Chemical characterization of fine particulate matter, source apportionment and long-range transport clusters in Thohoyandou, South Africa. Clean Air J. 2020;30(2):1–12. 10.17159/caj/2020/30/2.8735

[67] Szewc K, Graca B, Dołęga A. Atmospheric deposition of microplastics in the coastal zone: Characteristics and relationship with meteorological factors. Sci Total Environ. 2021;761:143272. doi: 10.1016/j.scitotenv.2020.143272 33162128

[68] Roblin B, Ryan M, Vreugdenhil A, Aherne J. Ambient atmospheric deposition of anthropogenic microfibers and microplastics on the western periphery of Europe (Ireland). Environ Sci Technol. 2020;54(18):11100–8. doi: 10.1021/acs.est.0c04000 32790996

