# Supplementary material for: Atmospheric deposition of microplastics in urban, rural, forest environments: A case study of Thulamela Local Municipality
Source: PLoS One. 2025 Mar 3;20(3):e0313840. doi: 10.1371/journal.pone.0313840 (PMC11875349; doi:10.1371/journal.pone.0313840)
Supplement: S1 Table — (DOCX) [file pone.0313840.s001.docx]

S1 Table. Microplastic deposition fluxes across three sampling environments over time (6 weeks).

| Sampling weeks |  | MP items/m^2^/day | |
| --- | --- | --- | --- |
|  | Urban | Rural | Forest |
| Week 1 | 324.1 | 117.9 | 62.3 |
| Week 2 | 459.6 | 189.2 | 62.4 |
| Week 3 | 196.3 | 156.0 | 52.7 |
| Week 4 | 270.7 | 187.9 | 97.1 |
| Week 5 | 374.7 | 248.5 | 130.8 |
| Week 6 | 508.5 | 237.3 | 137.8 |
| Mean ± SD | 355.6±47.6 | 189.5±20.0 | 90.5±15.2 |
