# Supplementary material for: Atmospheric deposition of microplastics in urban, rural, forest environments: A case study of Thulamela Local Municipality
Source: PLoS One. 2025 Mar 3;20(3):e0313840. doi: 10.1371/journal.pone.0313840 (PMC11875349; doi:10.1371/journal.pone.0313840)
Supplement: S2 Table — (DOCX) [file pone.0313840.s002.docx]

S2 Table. Overall colour, shape, polymer and size proportions (%) across urban, rural and forest environments over time (6 weeks).

| Colour | Proportion | Shape | Proportion | Polymer | Proportion | Size (μm) | Proportion |
| --- | --- | --- | --- | --- | --- | --- | --- |
| Black | 5.2 | **Fibre** | 89.2 | **PA** | 0.9 | **1000** | 13.7 |
| Blue | 4.8 | **Film** | 8.7 | **PE** | 27.2 | **500** | 13.6 |
| Green | 1.6 | **Foam** | 1.6 | **PET** | 42.3 | **250** | 25.5 |
| Red | 5.0 | **Fragment** | 0.5 | **PP** | 17.2 | **100** | 47.2 |
| Transparent | 52.4 |  |  | **PS** | 10.6 |  |  |
| White | 27.8 |  |  | **PVA** | 0.6 |  |  |
| Yellow | 3.1 |  |  | **PVC** | 1.2 |  |  |
