# Supplementary material for: Atmospheric deposition of microplastics in urban, rural, forest environments: A case study of Thulamela Local Municipality
Source: PLoS One. 2025 Mar 3;20(3):e0313840. doi: 10.1371/journal.pone.0313840 (PMC11875349; doi:10.1371/journal.pone.0313840)
Supplement: S3 Table — (DOCX) [file pone.0313840.s003.docx]

S3 Table. Range of meteorological variables measured across three sampling environments over time (6 weeks).

| Variables | Units | Week 1 | Week 2 | Week 3 | Week 4 | Week 5 | Week 6 | |
| --- | --- | --- | --- | --- | --- | --- | --- | --- |
| Humidity | % | 45 – 95 | 50 – 90 | 55 - 95 | 45 – 85 | 40 – 65 | 50 – 90 | |
| Temperature | °C | 23 – 24 | 22.5 – 23.5 | 18.5 – 22 | 19 – 21.5 | 24.5 – 26.5 | | 19 – 22 |
| Windspeed | Km/h | 9 – 19 | 9 – 19 | 9 – 19 | 9 – 9 | 0 – 19 | 9 – 19 | |
| Rainfall | mm | 3 – 3 | 3 – 5 | 0 – 0 | 5 – 15 | 0 – 0 | 3 – 39 | |
